# Prediction of multivessel coronary artery disease and candidates for stress-only imaging using multivariable models with myocardial perfusion imaging

**DOI:** 10.1007/s12149-022-01751-7

**Published:** 2022-06-05

**Authors:** Yuji Kunita, Kenichi Nakajima, Tomoaki Nakata, Takashi Kudo, Seigo Kinuya

**Affiliations:** 1 Department of Nuclear Medicine, Kanazawa University Hospital, Kanazawa, Japan; 2 Department of Functional Imaging and Artificial Intelligence, Kanazawa University, Kanazawa, Japan; 3 Department of Cardiovascular Medicine, Hakodate Goryoukaku Hospital, Hakodate, Japan; 4 Department of Radioisotope Medicine, Atomic Bomb Disease and Hibakusha Medicine Unit, Atomic Bomb Disease Institute, Nagasaki University, Nagasaki, Japan

**Keywords:** Coronary artery disease, Single-photon emission computed tomography, Multivariable model, Quantitation

## Abstract

**Purpose:**

Selecting patients with coronary multivessel disease (MVD) or no stenosis using myocardial perfusion imaging (MPI) is challenging. We aimed to create a model to predict MVD using a combination of quantitative MPI values and background factors of patients. We also assessed whether patients in the same database could be selected who do not require rest studies (stress-only imaging).

**Methods:**

We analyzed data from 1001 patients who had been assessed by stress MPI at 12 centers and 463 patients who had not undergone revascularization in Japan. Quantitative values based on MPI were obtained using cardioREPO software, which included myocardial perfusion defect scores, left ventricular ejection fractions and volumes. Factors in MPI and clinical backgrounds that could predict MVD were investigated using univariate and multivariate analyses. We also investigated whether stress data alone could predict patients without coronary stenosis to identify candidates for stress-only imaging.

**Results:**

We selected summed stress score (SSS), rest end-diastolic volume, and hypertension to create a predictive model for MVD. A logistic regression model was created with an area under the receiver operating characteristics curve (AUC) of 0.825. To more specifically predict coronary three-vessel disease, the AUC was 0.847 when SSS, diabetes, and hypertension were selected. The mean probabilities of abnormality based on the MVD prediction model were 12%, 24%, 40%, and 51% for no-, one-, two-, and three-vessel disease, respectively (*p* < 0.0001). For the model to select patients with stress-only imaging, the AUC was 0.78 when the model was created using SSS, stress end-systolic volume and the number of risk factors (diabetes, hypertension, chronic kidney disease, and a history of smoking).

**Conclusion:**

A model analysis combining myocardial SPECT and clinical data can predict MVD, and can select patients for stress-only tests. Our models should prove useful for clinical applications.

## Introduction

Visual evaluation among nuclear cardiology examinations is the first-line assessment of myocardial perfusion defects and abnormalities. However, quantitative evaluations using indices calculated from ungated and gated myocardial perfusion imaging (MPI) have recently become prevalent and are used to treat coronary artery disease (CAD) [1]. Quantitative analyses using dedicated software and defect scoring have become popular for diagnosing myocardial ischemia, as well as ischemia or infarction from stress and rest data. These quantitative methods are effective for diagnostic and prognostic evaluation [2–4].

However, conventional single-photon emission computed tomography (SPECT) can accurately detect localized ischemia of one-vessel disease, but its ability to estimate multivessel (MVD), especially three-vessel (3VD) disease is limited. That is, even in patients with MVD, only an area with the most severe stenosis might be detected as a culprit lesion, or the phenomenon of balanced ischemia could result in no discernible perfusion defect. One way to compensate for this is to detect left ventricular functional abnormalities after stress tests, such as transient ischemic dilation and reduced left ventricular contractility after stress [5–7]. However, how to integrate and apply these data has not been investigated in detail.

Stress-only tests have been recommended to reduce the radiation dose and frequency of SPECT examinations [8]. However, how to select patients who do not require rest tests is also important to consider in clinical practice.

Here, we aimed to create models with which to predict patients with MVD and to select patients who need only stress tests based on their background and quantitative values derived from myocardial perfusion SPECT images.

## Methods

### Demographics

We selected data from a multicenter database of 1001 patients who had been evaluated by MPI at 12 centers in Japan. Our previous findings using this database are published elsewhere [9, 10]. The database includes age, sex, height, weight, gated SPECT data such as summed stress/rest/difference scores (SSS/SRS/SDS), left ventricular ejection fraction (EF), end-diastolic and end-systolic volumes (EDV and ESV, respectively), risk factors of CAD, degree of coronary artery narrowing on selective coronary angiography (CAG) or coronary CT angiography (CCTA), and a history of percutaneous coronary intervention (PCI) or coronary artery bypass grafting (CABG). Data based on gated SPECT were analyzed using cardioREPO software, which we developed in collaboration with FUJIFILM Toyama Chemical, Co, Ltd. (Tokyo, Japan) and EXINI Diagnostics (Lund, Sweden).

The database consisted of 1,001 patients (male, *n* = 750; and female, *n* = 251), with a mean age of 69 ± 10 (range 21–98) years (Table 1). To increase the reliability of detecting CAD, we excluded 430 patients who had undergone coronary revascularization and 108 with no vessel disease (0VD) despite previous myocardial infarction with SRS ≥ 7. The latter patients were described as 0VD based on the latest CAG findings after coronary revascularization. We finally analyzed data from 463 patients of whom 324 (70%) were male.

**Table 1:** Demographics of databases and selected patients

	All (*n* = 1001)		No revascularization (*n* = 463)	
Parameter	Mean (*n*)	SD	Mean (*n*)	SD
Age (years)	69	10	70	10
Sex (male %)	750	75%	324	70%
Height (cm)	162	9.1	162	9.3
Weight (kg)	63	13	63	13
Body mass index (kg/m^2^)	24	3.8	24	3.8
Summed stress score	9.5	9.9	6.9	8.2
Summed rest score	7	8.6	4.5	7.2
Summed difference score	3.3	3.9	2.9	3.2
Stress end-diastolic volume	106	40	101	38
Stress ejection fraction	65	13	67	12
Stress end-systolic volume	40	30	36	27
Rest end-diastolic volume	105	38	101	36
Rest ejection fraction	67	13	68	12
Rest end-systolic volume	37	29	34	26
Stress/rest EDV ratio	1	0.096	1	0.1
Stress–Rest ejection fraction	− 1.8	6	− 1.9	6.4
Diabetes mellitus	381	47%	158	45%
Hypertension	606	73%	275	73%
Dyslipidemia	505	65%	210	64%
CKD (eGFR < 60)	246	32%	132	34%
Hemodialysis/CAP dialysis	29	3.80%	14	4%
Current smoking	154	23%	59	24%
History of smoking	261	41%	99	46%
Angina pectoris	236	37%	86	39%
History of myocardial infarction	205	27%	75	16%

Data are shown as means with standard deviations (SD) or as ratios (%)

*CAP* continuous ambulatory peritoneal, *CKD* chronic kidney disease

Based on non-gated SPECT data, the means of SSS, SRS, and SDS, were 6.9 ± 8.2, 4.5 ± 7.2, and 2.9 ± 3.2. Gated SPECT data were calculated using cardioREPO software (FUJIFILM Toyama Chemical Co. Ltd., and EXINI Diagnostics AB). The left ventricular functional parameters were as follows: EDV at stress (sEDV) and at rest (rEDV), 101 ± 38 and 101 ± 36 mL, respectively; ESV at stress (sESV) and at rest (rESV), 36 ± 27 and 34 ± 26 mL, respectively; and EF at stress (sEF) and at rest (rEF), 67 ± 12 and 68 ± 12%, respectively.

The ratios of comorbidities were 45%, 73%, 64%, 34%, and 16% for diabetes mellitus (DM), hypertension (HT), dyslipidemia (DL), chronic kidney disease (CKD), and old myocardial infarction OMI.

### Definition of coronary artery disease

We diagnosed CAD based on the American Heart Association (AHA) definition as significant (≥ 75%) stenosis of the coronary artery on coronary angiograms.

### Myocardial perfusion imaging

Patients were assessed using a 1-day protocol of MPI with standard exercise (37%) or with pharmacological (63%, adenosine, 120 μg/mL × 6 min) stress and SPECT at the participating hospitals. All these institutions used ^99m^Tc-labeled hexakis-2-methoxyisobutylisonitrile (MIBI), with a second injection dose that was 2–threefold more than the first dose. The total dose was 740–1110 MBq.

### SPECT data acquisition and processing

SPECT data were acquired using a standard image acquisition protocol in each hospital [11, 12], but the precise methods were not regulated. The manufacturers of the SPECT equipment included Siemens, GE, Philips, Hitachi, and Picker companies. The energy setting was centered at 140 keV with a 15–20% window). Collimators were either low-energy high-resolution or cardiac high-resolution types. SPECT imaging duration ranged from 20 to 50 s per projection, and projection images were collected in a 64 $\times$ 64 matrix. SPECT collection step angles ranged from 5° to 9°, with a rotational range of 180° or 360°. The ECG gating of the dual-head SPECT system was 16 or 8 frames per cardiac cycle. SPECT data were reconstructed using a filtered back projection method but one institution used a maximum-likelihood expectation maximization method. Attenuation and scatter correction were not applied. The image quality of the SEPCT data was confirmed in a core laboratory.

### Artificial neural network

Left ventricular function was analyzed throughout the study using cardioREPO as described [9, 13]. Briefly, we determined the shape of the entire left ventricle using an active-shape model for left ventricular contour extraction. After extracting the contours of candidate regions with low accumulation, the probability of anomalies was determined using an artificial neural network (ANN). The method is based on features such as shape, extent, location, number, perfusion uniformity, local motion, wall thickening, and sex to comprehensively determine the presence or absence of ischemia as in clinical human diagnosis. The ANN was trained on a multicenter database of ^99m^Tc-MIBI myocardial perfusion SPECT data derived from 1001 patients, and on interpretations by nuclear cardiology specialists.

### Defect scoring

We assessed SPECT images by dividing the entire left ventricular myocardium into 17 segments, then scoring each segment from normal to complete defects as 0–5 and calculating total scores. The summed deficit scores in stress and rest segments on images were defined as SSS and SRS, respectively. Thereafter, cardioREPO automatically calculated SDS (ischemia scores) by subtracting the SRS from the SSS for each segment. A normal MPI database (JSNM standard) [14] created by a working group of Japanese Society of Nuclear Medicine (JSNM) is included in cardioREPO.

### Statistics

Data are presented as means ± standard deviation (SD). Groups were compared using one-way analysis of variance (ANOVA) and *t* tests. Explanatory variables were analyzed using univariable and multivariable logistic regression models and receiver operating characteristics (ROC) analysis with areas under the ROC curves (AUC). The cutoff of the variables was set to the value that maximized (sensitivity + specificity − 1). Valid variables with < 0.10 in the univariate analyses were entered into a multivariate logistic regression analysis. Anomaly probabilities were calculated based on the variables as:

$$P\left( \% \right) \, = \, 100/(1 \, + {\text{ Exp}}\left[ { - \left( {b_{0} + \Sigma b_{i} X_{i})} \right]} \right),$$

using JMP v. 14 (SAS Institute Inc., Cary, NC, USA) statistical software. Values with *P* < 0.05 were considered statistically significant.

## Results

### Univariate analysis to predict MVD

Coronary stenosis was estimated by logistic regression analysis including all variables and MVD was defined as coronary two- or three-vessel disease (Table 2). The results for SSS, SRS, SDS, sEDV, sEF, sESV, rEDV, rEF, and rESV based on non-gated and gated SPECT were significant. Among the factors associated with disease states, DM, HT, DL, CKD (defined as eGFR < 60 mL/min/1.73 m^2^), smoking history, angina pectoris (AP), and OMI were significant. However, neither of the sEDV/rEDV and sESV/rESV ratios that correspond to transient ischemic dilation, was significant.

**Table 2:** Logistic regression analysis to predict multivessel coronary artery disease

Parameter	*χ*^2^	*p*	Unit OR	AUC
Age (y)	0.18	0.668	1	0.532
Sex (male)	15.22	< 0.0001	3.33	0.605
Height (cm)	6.11	0.014	1.03	0.586
Weight (kg)	3.67	0.056	1.02	0.573
Body mass index (kg/m^2^)	0.58	0.446	1.02	0.535
Summed stress score	71.49	< 0.0001	1.16	0.793
Summed rest score	57.31	< 0.0001	1.19	0.762
Summed difference score	26.82	< 0.0001	1.19	0.699
Stress end-diastolic volume	46.49	< 0.0001	1.02	0.732
Stress ejection fraction	37.94	< 0.0001	0.94	0.682
Stress end-systolic volume	44.45	< 0.0001	1.03	0.739
Rest end-diastolic volume	44.65	< 0.0001	1.02	0.729
Rest ejection fraction	34.75	< 0.0001	0.94	0.668
Rest end-systolic volume	38.34	< 0.0001	1.03	0.733
Stress/rest EDV ratio	1.42	0.234	3.41	0.53
Stress-Rest ejection fraction	0.71	0.401	0.99	0.548
Diabetes mellitus	17.57	< 0.0001	3.07	0.636
Hypertension	7.98	0.005	2.59	0.581
Dyslipidemia	11.78	0.001	3.03	0.612
CKD (eGFR < 60)	6.52	0.011	1.88	0.574
Hemodialysis/CAP dialysis	0.57	0.452	1.58	0.511
Current smoking	1.47	0.225	1.51	0.539
Past smoking	11.8	0.001	3.41	0.647
Angina pectoris	8.43	0.004	3.06	0.636
History of myocardial infarction	86.79	< 0.0001	15.28	0.723

*AUC* area under the receiver operator characteristics, *CAP* continuous ambulatory peritoneal, *CKD* chronic kidney disease, *OR* odds ratio

### Univariate analysis to predict 3VD

Three-vessel disease was also estimated using logistic regression analysis (Table 3).

**Table 3:** Logistic regression analysis to predict three-vessel coronary artery disease

Parameter	*χ*^2^	*p*	Unit OR
Age (y)	0.11	0.738	1.01
Sex (male)	8.47	0.004	3.67
Height (cm)	2.79	0.095	1.03
Weight (kg)	4.2	0.04	1.02
Body mass index (kg/m^2^)	2.52	0.113	1.06
Summed stress score	56.64	< 0.0001	1.13
Summed rest score	45.62	< 0.0001	1.13
Summed difference score	22.88	< 0.0001	1.2
Stress end-diastolic volume	22.09	< 0.0001	1.02
Stress ejection fraction	21.37	< 0.0001	0.95
Stress end-systolic volume	23.28	< 0.0001	1.02
Rest end-diastolic volume	22.37	< 0.0001	1.02
Rest ejection fraction	20.3	< 0.0001	0.95
Rest end-systolic volume	20.97	< 0.0001	1.02
Stress/rest EDV ratio	0.09	0.768	1.5
Stress–rest ejection fraction	0.23	0.633	0.99
Diabetes mellitus	21.45	< 0.0001	8.36
Hypertension	7.23	0.007	5.18
Dyslipidemia	7.11	0.008	3.42
CKD (eGFR < 60)	3.89	0.049	1.89
Hemodialysis/CAP dialysis	2.25	0.134	2.78
Current smoking	1.2	0.274	1.61
Past smoking	8.12	0.004	4.52
Angina pectoris	6.85	0.009	5.83
History of MI	63.87	< 0.0001	13.69

*CAP* continuous ambulatory peritoneal, *CKD* chronic kidney disease, *eGFR* estimated glomerular filtration rate, *MI* myocardial infarction, *OR* odds ratio

Volume, disease status, and smoking history were significantly associated with defect score and cardiac function like MVD, whereas the sEDV/rEDV and sESV/rESV ratios were not.

### Comparisons among groups with and without coronary stenosis

We compared variables for no-, one-, two-, and three- vessel disease (0, 1, 2, and 3VD, respectively) using one-way ANOVA (Table 4).

**Table 4:** Comparison among groups with 0, 1, 2, and 3 vessel diseases

Number of stenosis	0VD		1VD		2VD		3VD		
Items	Mean	SD	Mean	SD	Mean	SD	Mean	SD	P*
Age (y)	69.4	10.6	71	9.7	68.4	9.1	70.1	7.7	0.5001
Sex (male)	178	62%	58	81%	42	84%	46	88%	< 0.0001
Height (cm)	160.4	9.6	162.5	9	163.5	8.7	163.6	8.3	0.0272
Weight (kg)	61.7	13.2	62.7	12.6	63.4	13.4	66.1	11.3	0.1615
Body mass index (kg/m^2^)	23.9	4	23.6	3.4	23.6	3.9	24.7	3.6	0.3996
Summed stress score	3.8	3.6	8.2	8.6	12.2	9.5	17.3	12.4	< 0.0001
Summed rest score	1.9	1.8	5.6	7.7	9.1	9.6	13.1	12	< 0.0001
Summed difference score	2.3	2.7	3.2	3.6	3.9	3.3	5.2	4	< 0.0001
Stress EDV	90.6	28.5	105.6	37.9	127	47.4	125.8	45.9	< 0.0001
Stress EF	69.1	10	66.2	11.5	60.1	15.3	58.8	14.6	< 0.0001
Stress ESV	28.5	15.1	38.3	28.4	55.6	42.4	55.3	35.2	< 0.0001
Rest EDV	91.1	27.8	106	37.4	124.7	44.2	124.9	43.2	< 0.0001
Rest EF	70.8	9.4	68.2	11.7	62.6	15.6	61.1	14.3	< 0.0001
Rest ESV	27.3	15.1	36.2	28.3	51.8	41.6	52.1	34.7	< 0.0001
Stress/rest EDV ratio	1	0.1	1	0.11	1	0.11	1	0.1	0.5268
Stress–rest EF	− 1.7	6.8	− 2	5.2	− 2.4	5.7	− 2.3	6.6	0.8283
Diabetes mellitus	78	38%	28	41%	19	48%	33	85%	< 0.0001
Hypertension	155	69%	50	70%	33	79%	37	93%	0.0149
Dyslipidemia	113	57%	37	66%	28	78%	32	84%	0.0022
CKD (eGFR < 60)	67	30%	24	34%	20	43%	21	48%	0.0669
HD/CAP dialysis	6	3%	4	6%	1	3%	3	10%	0.3213
Current smoking	33	21%	9	25%	8	28%	9	32%	0.5996
Past smoking	48	34%	19	70%	15	63%	17	77%	< 0.0001
Angina pectoris	45	31%	20	48%	11	52%	10	77%	0.0019
History of MI	0	0%	23	32%	20	40%	32	62%	< 0.0001

Data are shown as means with standard deviations (SD) or as ratios (%)

*CAP* continuous ambulatory peritoneal, *CKD* chronic kidney disease, *EDV* end-diastolic volume, *EF* ejection fraction, *ESV* end-systolic volume, *eGFR* estimated glomerular filtration rate, *HD* hemodialysis, *MI* myocardial infarction, *VD* vessel disease

*Pearson statistics

Differences among groups and factors associated with comorbidities were significant, whereas CKD and sEDV/rEDV were not.

### Multivariate analysis to predict MVD

**Fig. 1:**
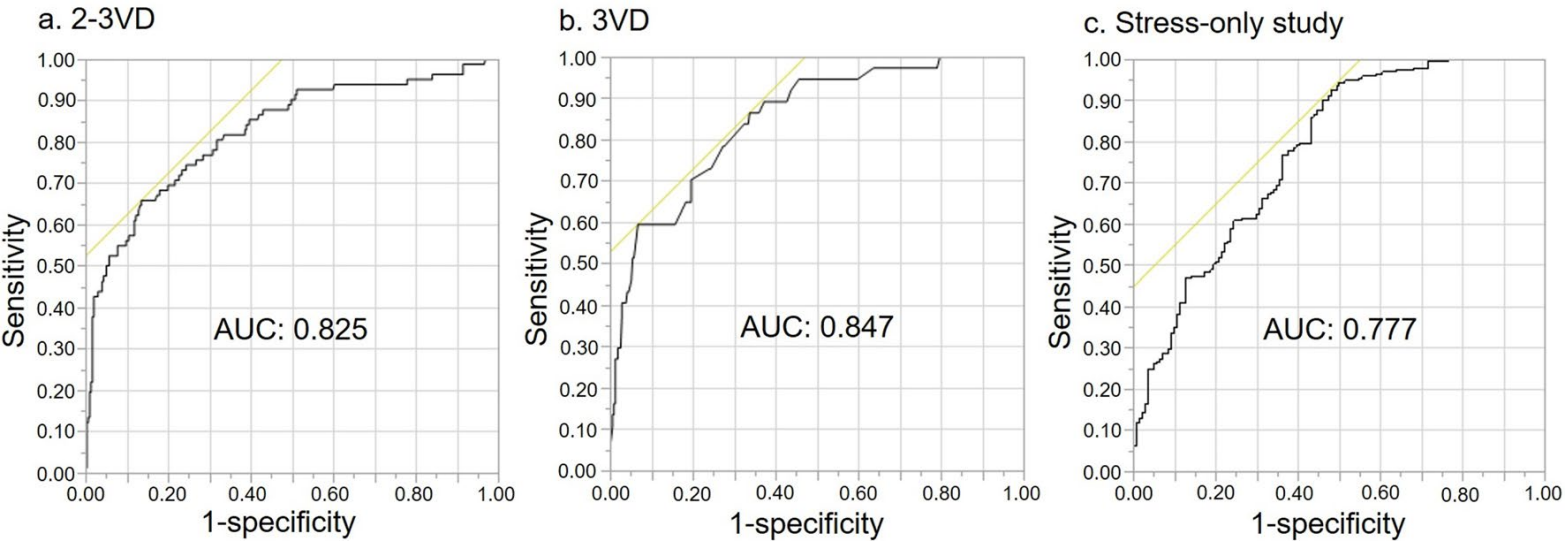
Receiver operating characteristics (ROC) curves of MVD (**a**), 3VD (**b**), and 0VD (**c**) prediction models. The sensitivity and specificity for each were 66% and 86% (**a**), 86% and 66% (**b**), and 93% and 52% (**c**), respectively. *0VD* no vessel disease, *3VD* three-vessel disease, *MVD* multivessel disease

We attempted to create a predictive model for MVD by selecting clinically generalizable items that significantly differed (*p* < 0.1). Multivariate stepwise regression selected SSS, rEDV, and HT that might predict MVD. Table 5 shows estimates of these parameters, and the AUC was 0.825 (Fig. 1).

**Table 5:** Multivariable logistic analysis to predict multivessel, three-vessel and zero-vessel disease

Parameter	Estimated value	Standard error	*χ*^2^	*p*	Unit OR
Multi-vessel disease					
Intercept	− 4.15	0.59	49.5	< 0.0001	
SSS	0.14	0.022	37.2	< .0001	1.14
Rest EDV	0.010	0.0050	5.00	0.025	1.01
Hypertension	0.89	0.38	5.37	0.021	2.43
3-vessel disease					
Intercept	− 5.25	0.75	49.0	< 0.0001	
SSS	0.099	0.020	23.9	< 0.0001	1.10
Diabetes mellitus	1.57	0.49	10.5	0.0012	4.81
Hypertension	1.46	0.66	4.95	0.026	4.32
0-vessel disease*					
Intercept	2.29	0.33	49.1	< 0.0001	
SSS	− 0.16	0.029	30.9	< 0.0001	0.85
Stress ESV	− 0.015	0.0079	3.50	0.061	0.99
Multiple risk factors (*n*)	− 0.27	0.13	4.56	0.033	0.76

*EDV* end-diastolic volume, *OR* odds ratio, *SSS* summed stress score

*Only stress data and clinical variables are included

### Multivariate analysis to predict 3VD

We created a model to predict 3VD by including SSS, DM, and HT in the multivariate analysis. Table 5 shows the parameter estimates and the AUC was 0.847 (Fig. 1).

### Probability of MVD

We calculated the probability of 0, 1, 2, and 3VD using the model:

$$\begin{aligned} {\text{logit}}\left[ {{\text{MVD}}} \right] \, = & \, - {4}.{15 } + \, 0.{135 } \times {\text{ SSS}} \\ & + \, 0.0{1}0{4 } \times {\text{ rest EDV }}\left( {{\text{mL}}} \right) \, \\ & + \, 0.{887 } \times {\text{ HT }}\left( {1/{0 } = {\text{ yes}}/{\text{no}}} \right). \\ \end{aligned}$$

**Fig. 2:**
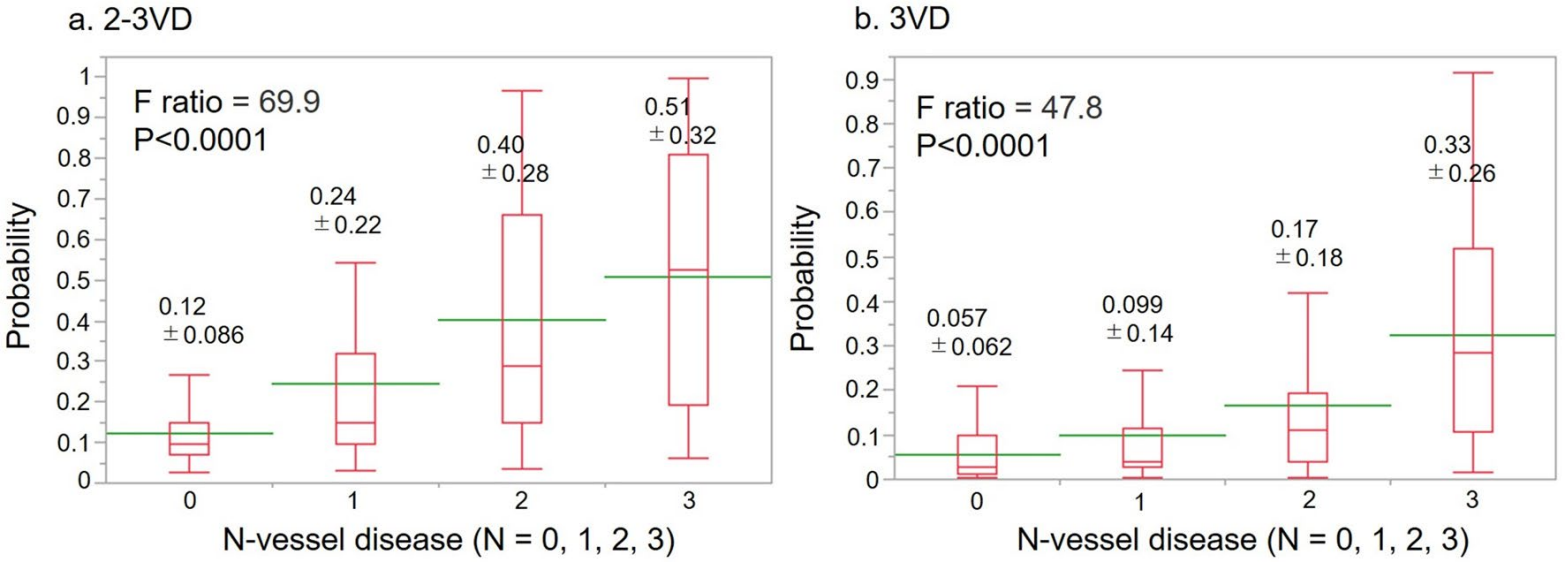
Comparison of probability of CAD using MVD (**a**) and 3VD (**b**) prediction models. The former and latter can, respectively, predict MVD and 3VD. *0VD* no vessel disease, *3VD* three-vessel disease, *MVD* multivessel disease

The average probabilities of abnormality in the MVD prediction model were 12%, 24%, 40%, and 51% for 0, 1, 2, and 3VD, respectively (*p* < 0.0001; Fig. 2).

### Probability of 3VD

The probability of 3VD was calculated using the model:

$$\begin{aligned} {\text{logit}}\left[ {{\text{3VD}}} \right] \, = & \, - {5}.{25 } + 0.0{993 } \times {\text{ SSS }} \\ & + { 1}.{57 } \times {\text{ DM }}\left( {1/{0 } = {\text{ yes}}/{\text{no}}} \right) \, \\ & + { 1}.{46 } \times {\text{ HT }}\left( {1/{0 } = {\text{ yes}}/{\text{no}}} \right). \\ \end{aligned}$$

A comparison of 0, 1, 2, and 3VD using this model revealed that the probabilities of abnormalities were 5.7%, 9.9%, 17.0%, and 33.0%, respectively (*p* < 0.0001; Fig. 2).

### MVD vs. 3VD prediction models

**Fig. 3:**
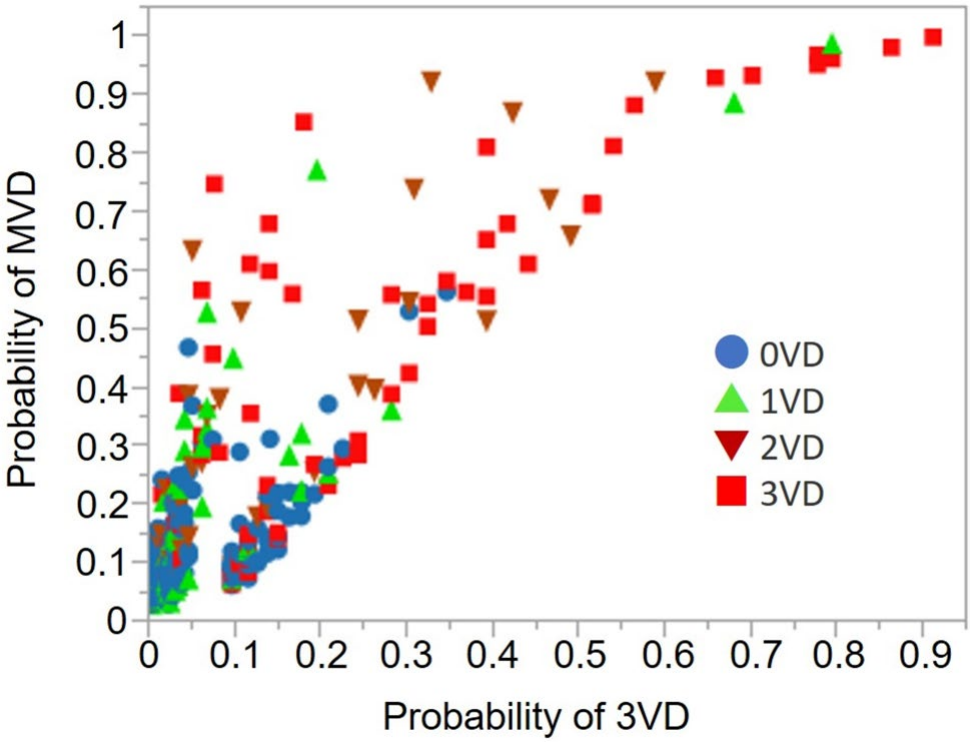
Comparison of MVD and 3VD prediction models. Probability in MVD and 3VD prediction models is shown in vertical and horizontal axes, respectively. Blue circles, green triangles, brown triangles, and red squares represent 0VD, 1VD, 2VD, and 3VD, respectively. *MVD* multivessel disease, *VD* vessel disease

We compared the two models using a bivariate analysis to determine which was more appropriate (Fig. 3). The predicted likelihood was higher, and the statistical significance of differences among groups was relatively higher for the MVD, than the 3VD model.

### Prediction of 0VD for stress-only imaging

We investigated whether MPI with exercise or adenosine stress alone can predict 0VD, that is, whether stress-only imaging can exclude significant coronary stenosis. We created a prediction model for 0VD based on quantitative values obtained from stress myocardial perfusion SPECT and patient background factors. The results of the stepwise regression showed that SSS contributed most to the creation of an accurate prediction model (AUC, 0.755), followed by sESV (AUC, 0.699) and the accumulation of multiple risk factors (AUC, 0.657). We calculated the number of risk factors using the sum of the factors (0–4) DM, HT, CKD, and a history of smoking.

The AUC of the model with SSS plus sESV was 0.759, and slightly improved over SSS alone. Adding more risk factors to SSS and sESV resulted in a more accurate prediction model with an AUC of 0.777 (Fig. 1).

## Discussion

Although detecting MVD using MPI has been limited, the present study revealed that the probability of MVD can be estimated using a model that combines quantitative information from myocardial perfusion SPECT and the backgrounds of patients. Our model selected candidates appropriate for stress-only imaging,

### Application of SPECT for MVD

Several reasons have been postulated to explain the limited ability of SPECT to diagnose MVD [15]. One is that 75% coronary artery stenosis might not result in significantly reduced perfusion. Furthermore, linearity between true myocardial blood flow and myocardial accumulation has been considered insufficient with single-photon radiopharmaceuticals [16, 17]. Lesions with the worst stenosis might be detected, whereas less severe areas might be overlooked, and 3VD might be undetectable due to balanced ischemia [18, 19], which is a limitation of SPECT based on relative count distribution. Therefore, we considered that myocardial perfusion combined with left ventricular function and patient background could predict MVD. We confirmed that a judgement of myocardial defects was insufficient, but adding cardiac function and clinical background surpassed the perfusion-only method. In fact, DM, HT and smoking were factors associated with CAD; thus having more than one factor might indicate a higher likelihood of CAD as shown in our models. We did not include DL in the list of multiple factors, because we found that the possibility of CAD was statistically lower when DL was included. This might be associated with the fact that the prevalent treatment of DL with statins also reduces the risk of all-cause mortality in patients with a medical history of CAD [20, 21].

### Stress-only imaging

The stress-only SPECT concept is becoming widespread worldwide, because the burden on patients can be reduced by not having to endure the rest test when stress myocardial perfusion scintigraphy clearly shows no possibility of CAD [5, 6, 22]. This approach is also recommended by the International Atomic Energy Agency Nuclear Cardiology Protocols Study (INCAPS) [8]. However, stress tests under low-risk conditions have not been routinely applied in Japan. The conventional method of visual assessment based only on stress perfusion defects might not correctly assess CAD and overlook patients who should actually be indicated for further coronary artery assessments. Therefore, we also investigated whether myocardial perfusion, left ventricular function, and the backgrounds of patients can be used to discriminate candidates for stress-only imaging.

### Selection of patients from the multicenter database

Quantitative and clinical data from a multicenter database were analyzed to create a model to predict MVD. We used information collected from several centers where patient background factors including the presence of CAD and myocardial perfusion were complete. Therefore, the selection of patients might have been more heterogeneous than in single-center studies. However, that the database reflected the average patient population indicated for MPI studies could be an advantage in Japan.

We excluded patients who had been treated by coronary revascularization. One reason was that post-treatment status was too confusing to accurately assess, even with contemporary CAG. Another reason was that such clinical predictions of MVD do not apply to patients after revascularization. Although the model was based on patients selected under these conditions, we generated accurate models with AUCs of 0.825 and 0.847 for MVD and 3VD, respectively, and the specificity and sensitivity of the models were both ~ 80%.

### Comparison with conventional MVD detection

The indicators of high risk include MVD and left main trunk disease, decreased wall motion representing stunned myocardium with stress, LVEF < 45% at rest or stress, a decrease in LVEF of ≥ 5% after stress, left ventricular transient ischemic dilation (TID; ventricular cavitary enlargement > 10% compared with resting state), increased radioisotope accumulation in the lung field, and right ventricular delineation [23–27]. However, even if one of these factors indicated the possibility of MVD, the probability remained unknown due to the limitations of single factor estimation. The present results quantified the likelihood of MVD, which might provide more specific information than conventional methods. The results of our multivariate analysis showed that indices equivalent to TID and findings of post-stress dysfunction such as decreased EF after stress, were not significant beyond the combination of SSS and ESV. This does not imply that post-stress dysfunction has no value for individual patients, but rather that comprehensive judgment is still needed.

### Roles of functional stress imaging and CCTA

In addition to MPI, coronary CT angiography (CCTA) is becoming more popular in routine clinical practice to non-invasively assess coronary arteries. Although CCTA provides a good morphological assessment of coronary artery stenosis and plaque, it is not necessarily suitable for evaluating actual myocardial ischemia [28–30]. In addition, CCTA might not be sufficient for patients with arrhythmia and/or severe calcification, and the side effects of radiation exposure, and the effects of contrast media in patients with CKD need to be understood and considered. However, both MPI and CCTA are useful tests for diagnosing CAD, and complementary roles for the diagnosis of CAD should be emphasized [31]. In general, CCTA and MPI are often the imaging modalities of choice for predicting the pre-test likelihood of moderate CAD. Therefore, if patients are initially assessed using MPI, the probability of MVD will help to select subsequent diagnostic procedures. If patients initially assessed using CCTA have no obvious stenosis, the possibility of CAD is considered low. However, when the culprit coronary arteries and the degree of ischemia cannot be fully evaluated because of issues such as calcification, or several coronary arteries with similar degrees of stenosis, SPECT should be considered to estimate the possibility of MVD from the viewpoint of perfusion and function.

Japanese Circulation Society’s guideline focused update (2022) on diagnosis and treatment in patients with stable CAD advocate the use of functional stress imaging and CCTA as follows [32]. In institutions where a CT scanner is the only imaging device, it is suggested that CCTA first rule out non-obstructive coronary artery. If the institution is experienced with functional stress imaging including myocardial SPECT, it is suitable to mainly apply those imaging techniques for diagnosis and risk stratification. In institutions capable of performing multimodal imaging, CCTA is the preferred imaging to rule out the presence of CAD. On the other hand, stress imaging is preferred as an initial imaging test in patients with a high pre-test probability or known history of CAD for risk assessment. This approach can be summarized as “rule-out dominant” and “rule-in dominant” strategies for CCTA and myocardial perfusion SPECT, respectively.

### Candidate patients for stress-only imaging

We also developed a model to predict candidates for stress-only imaging, and the AUC was ~ 0.8. Although the model can be clinically applied, the AUC indicated that some patients with coronary stenosis might be overlooked. When we confirmed stenosis in patients despite a high probability of 0VD, ischemia and post-stress dysfunction were not evident, and functional status was good. This could mean that the study included patients whose status had reached the limitations of SPECT imaging. Therefore, patients with significant anginal symptoms or suspected coronary stenosis based on overall clinical factors should be evaluated by stress and rest tests even if the model-based risk is low.

### Model-based approach

The prognostic value of MPI in the absence of ischemia has been confirmed by national and international multicenter studies [3, 4, 33, 34]. However, the model-based approach might enhance the possibility of MVD even in patients diagnosed with MPI in the absence of perfusion defects and ischemia, by combining cardiac function and clinical factors, indicating further examinations for CAD. Another possible option is to use a model for selecting patients for stress-only test by omitting the rest test. Ultimately, prognosis together with such predictions will require evaluation, but since the database did not include prognostic information, further studies will be required to determine the prognostic value of the model.

Whether or not MVD can be predicted by resting MPI alone needs to be discussed. First, a model including only resting MPI could be generated to predict MVD using quantitative values and clinical information obtained in the same manner as we analyzed in this study. However, we found that the SSS obtained by stress MPI was an indispensable factor for a highly accurate MVD prediction model. Since stress-induced ischemia has significant roles for the diagnosis of the severity of CAD and management strategy, the rest-only model might overlook the possibility of the MVD.

### Limitations

Patients undergoing revascularization were excluded, because CAD had already been evaluated, which limited the number of patients. Therefore, in principle, our findings should be applied to patients who have not been evaluated by CAG.

This study included SPECT and partial CCTA information without stress myocardial perfusion magnetic resonance imaging (MRI), which a noninvasive method of coronary artery evaluation. A recent meta-analysis of stress myocardial perfusion MRI with gadolinium contrast media and pharmacological stress with adenosine has shown that the mean diagnostic sensitivity and specificity for coronary stenosis were 91% and 81%, respectively [35].

In addition, although we evaluated the possibility of MVD, the model does not determine which coronary artery is significantly stenosed. Further study is needed to accumulate more data.

## Conclusion

Our predictive model created by combining myocardial SPECT and clinical information can predict MVD and should generate valuable additive information. We also created a model for selecting candidates for stress-only imaging. A database of many inter-institutional studies will be required to validate this model.
